# Genome-Wide Gene/Genome Dosage Imbalance Regulates Gene Expressions in Synthetic *Brassica napus* and Derivatives (AC, AAC, CCA, CCAA)

**DOI:** 10.3389/fpls.2016.01432

**Published:** 2016-09-23

**Authors:** Chen Tan, Qi Pan, Cheng Cui, Yi Xiang, Xianhong Ge, Zaiyun Li

**Affiliations:** ^1^National Key Lab of Crop Genetic Improvement, National Center of Oil Crop Improvement (Wuhan), College of Plant Science and Technology, Huazhong Agricultural UniversityWuhan, China; ^2^Crop Research Institute, Sichuan Academy of Agricultural SciencesChengdu, China

**Keywords:** dosage imbalance, gene expression, *cis*/*trans* effects, *Brassica*, polyploids

## Abstract

Gene/genome dosage balance is an essential evolutionary mechanism for organisms to ensure a normal function, but the underlying causes of dosage-imbalance regulation remain poorly understood. Herein, the serial *Brassica* hybrids/polyploids (AC, AAC, CCA, CCAA) with different copies of A and C subgenomes from the same two parents of *Brassica rapa* and *Brassica oleracea* were synthesized to investigate the effects of genome dosages on gene expressions and interactions by using RNA-Seq. The expression changes of A- and C-subgenome genes were consistent with dosage alterations. Dosage-dependent and -independent genes were grouped according to the correlations between dosage variations and gene expressions. Expression levels of dosage-dependent genes were strongly correlated with dosage changes and mainly contributed to dosage effects, while those of dosage-independent genes gave weak correlations with dosage variations and mostly facilitated dosage compensation. More protein–protein interactions were detected for dosage-independent genes than dosage-dependent ones, as predicted by the dosage balance hypothesis. Dosage-dependent genes more likely impacted the expressions by *trans* effects, whereas dosage-independent genes preferred to play by *cis* effects. Furthermore, dosage-dependent genes were mainly associated with the basic biological processes to maintain the stability of the growth and development, while dosage-independent genes were more enriched in the stress response related processes to accelerate adaptation. The present comprehensive analysis of gene expression dependent/independent on dosage alterations in *Brassica* polyploids provided new insights into gene/genome dosage-imbalance regulation of gene expressions.

## Introduction

Polyploidy, or WGD (Whole-genome duplication) occurs in more than 70% flowering plants (Wood et al., 2009), and is an important evolutionary process for plant speciation (Comai, 2005; Chen, 2007; Chen and Birchler, 2013). Recent studies have demonstrated that polyploid establishment is promoted during times of environmental stress, confirming polyploidy as a road toward evolutionary success rather than an evolutionary dead end (Vanneste et al., 2014). The evolutionary success is thought to be related with new genetic materials provided by their duplicated genomes, which increases biodiversity and novel phenotypes compared with diploid progenitors and then renders the driven evolution of ecological tolerances (Taylor and Raes, 2004; Fawcett et al., 2013). The increased novel phenotypes could be caused by dosage regulation (Birchler and Veitia, 2007, 2012). Changes in gene copy number generally lead to gene expression alterations (Tang and Amon, 2013), eventually leading to phenotypic alterations.

Gene dosage balance is critical for normal development and phenotypic characteristics, for gene dosage balance changes with different combinations of gene dosage, leading to gene expression alteration, protein complexes or networks variations (Birchler et al., 2005; Birchler and Veitia, 2012; Veitia and Potier, 2015). Birchler and colleagues (Birchler et al., 2005; Veitia et al., 2008) suggested that aneuploidy generally had larger changes in phenotypes than polyploidy probably because of dosage imbalance. Based on the gene balance hypothesis or the dosage balance hypothesis (Freeling and Thomas, 2006; Birchler and Veitia, 2012), the stoichiometric changes of macromolecular complexes affected the stability and interaction of a protein in a regulatory complex, leading to novel phenotypic eventually. In maize, phenotypic variations of plants with haploid plus a dosage series of chromosome arm were observed, which was caused by effects of genomic imbalance (Birchler and Veitia, 2012). Dosage effects and dosage compensation occurred as two types of dosage regulation in the expression if the dosage of a gene was changed (Guo et al., 1996). Gene dosage effects were often observed as the expression increased or decreased in proportion to the gene copy number changes, and genomic dosage in maize caused a proportional effect on heterosis which was subject to dosage effects (Yao et al., 2013). Many studies of dosage compensation from various organisms, especially *Drosophila* elucidated that gene expression was often dosage compensated (McAnally and Yampolsky, 2010; Zhang et al., 2010; Lundberg et al., 2012). Gene dosage balance was also one of the important factors for non-additive gene expressions widely observed in polyploids, although additive expression was the prevailing pattern (Yoo et al., 2014). In addition, *cis/trans* effects could regulate gene expression at the mRNA level (Rockman and Kruglyak, 2006; Williams et al., 2007; Yoo et al., 2014), and numerous *trans*-acting dosage effects on gene expression of aneuploids were revealed (Guo and Birchler, 1994).

In polyploids, some duplicated gene copies (homeologs) should be lost as a polyploid individual must balance the combined potential and challenge of having two or more genomes together (Yoo et al., 2014). Thus, gene expression changes would be caused by gene dosage balance alteration with different combinations of gene dosage. This view has many case supports (Thomas et al., 2006; Schnable et al., 2011; Wang et al., 2011; Liu et al., 2014). Recent works suggested that relative and absolute dosage constraints ruled the preservation or loss of the duplicated genes right after a polyploid event in *Arabidopsis* (Bekaert et al., 2011). In addition, the losses were nonrandom, and genes belonging to specific functional classes, such as ribosomal protein genes and transcription factors, were more often retained in duplicate (Birchler et al., 2005; Thomas et al., 2006; Birchler and Veitia, 2010; McGrath et al., 2014; Moghe et al., 2014), suggesting they were dosage-sensitive genes (Birchler et al., 2001; Thomas et al., 2006). They were also defined as dosage-dependent and/or dosage-independent expression genes (Birchler et al., 2001; Shi et al., 2015). Thus, dosage dependent/independent is an essential evolutionary mechanism that influences expression and the fate of duplicated genes. Although, most duplicate genes produced by WGD were quickly lost (Scannell et al., 2007), models of neo-functionalization (functional diversification) and sub-functionalization (partitioning and/or elaboration of the function between daughter copies) have been proposed to illustrate the fate of duplicated genes and to explain the advantages of WGD compared to diploid parents (Hahn, 2009; Ohno, 2013; Roulin et al., 2013). These mechanisms could be mixed at play. Bekaert et al. (2011) suggested that relative dosage might be important immediately after WGD, whereas sub-, neo-functionalization, and absolute dosage could be operating later in the process of evolution. It was suggested that dosage-dependent expression could maintain growth and developmental stability, whereas dosage-independent expression could facilitate functional divergence between homeologs during polyploid evolution (Shi et al., 2015). Whereas, Lloyd et al. (2014) found that meiotic genes which were involved in fundamental function often returned to a single copy following WGD. The role of dosage-balance influence in regulatory evolution remains poorly understood.

*Brassica napus* L. (AACC, 2n = 38) is an allotetraploid species formed through natural interspecific hybridization between *Brassica rapa* L. (AA, 2n = 20) and *Brassica oleracea* L. (CC, 2n = 18) approximately 7500 years ago (Chalhoub et al., 2014). Resynthesized *B. napus* at initial generations has been widely investigated for the changes at different levels of DNA sequences (Song et al., 1995), chromosomes and chromosomal recombination (Xiong et al., 2011), alternative splicing (AS) (Zhou, R. et al., 2011), proteome (Marmagne et al., 2010), and phenotypes (Gaeta et al., 2007). Although, these studies have provided many new insights into the genetic and genomic consequence of allopolyploidization in *B. napus*, we still know very little about dosage-balance regulation contributing to gene expression and evolution in *Brassica* polyploids, and the underlying causes of dosage dependent/independent genes were largely elusive. The studies to investigate this process were mainly limited to the model plant *Arabidopsis thaliana* and parented polyploids, and more surveys of different species would enable more comprehensive understanding. Recent completion of genome sequencing of *B. rapa* (Wang et al., 2011), *B. oleracea* (Liu et al., 2014; Parkin et al., 2014), and *B. napus* (Chalhoub et al., 2014) provides an opportunity to understand the complex genomes, and explore gene expressions under variable genome dosages.

In this study, the series of *Brassica* hybrids/polyploids that contained different dosages of A and C genomes contributed by the same two genotypes of *B. rapa* and *B. oleracea* were synthesized and analyzed by RNA-Seq to investigate the effects of genome dosages on gene expression and interaction. The genome-wide correlations between dosage variation and gene expressions were studied and dosage-dependent and -independent genes were grouped, with their roles in molecular function and biological pathways examined. Furthermore, we tested whether the *cis*/*trans*- regulation effects were correlated to dosage balance. The results might help to better understand how dosage imbalance affects global gene expression levels.

## Materials and methods

### Plant materials

**Figure 1:**
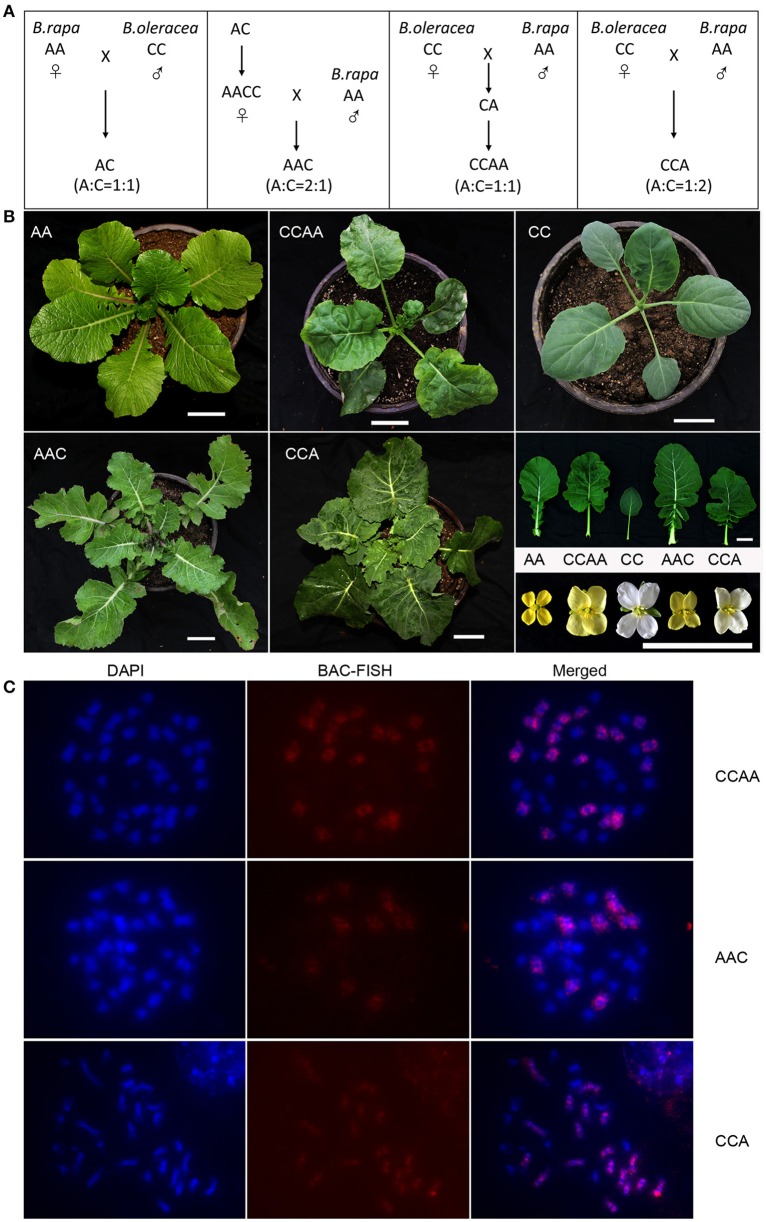
**Phenotype and cytology of synthetic *B. napus* and derivatives. (A)** Pedigrees of the plant materials. **(B)** Young plants, leaves and flowers of AA, CC, CCAA, AAC, and CCA. Scale bars = 5 cm. **(C)** DAPI, BAC-FISH, and merged images for each mitotic cell. Scale bars = 5 μm.

From reciprocal crosses between inbred lines of *B. rapa* L. (AA, 2n = 20, genotype 3H120) and *B. oleracea* L. (CC, 2n = 18, genotype Chijielan), *B. napus* F_1_ hybrid (AC, 2n = 19), allotetraploid (CCAA, 2n = 38) and triploid hybrid (CCA, 2n = 28) were produced (Cui et al., 2012) and used for this study, together with another triploid (AAC, 2n = 29) synthesized here. The allotetraploid CCAA originated directly from the cultured embryo-plantlet obtained from the *B. oleracea* × *B. rapa* cross without colchicine treatment, probably the spontaneous chromosome doubling occurred *in vitro* (Cui et al., 2012). The triploid hybrid CCA also from *B. oleracea* × *B. rapa* cross likely resulted from the fusion of unreduced gamete (CC) by the female parent *B. oleracea* and reduced gamete (A) by the male parent (Cui et al., 2012). The allotriploid (AAC) was produced by pollinating the clonal plants of the allotetraploid (AACC) with *B. rapa*, with the aid of immature embryo culture on MS agar medium without hormones (Murashige and Skoog, 1962; Figure 1A). All these materials were maintained and propagated by subculturing the young buds on MS medium with 1.5 mg/l^−1^ 6-benzyl aminopurine (6-BA) and 0.25 mg/l^−1^ α-naphthalenacetic acid (NAA), to produce enough plants for study, following the previous procedure (Cui et al., 2012). Plantlets grew on MS medium in the growth chamber at 25°C and a 14/10 h (day/night) photoperiod, and the newly emerged and expanded young leaves were collected and immediately frozen in liquid nitrogen for RNA extraction. We adopted a mixed sampling strategy with three plantlets of each material and two biological replicates for each sample.

### Fluorescence *in situ* hybridization (FISH)

Young ovaries were collected and treated with 2 mM 8-hydroxyquinoline for 3–4 h at 22°C, and subsequently fixed in Carnoy's solution I (3:1 ethanol: glacial acetic acid, v/v) for 24 h, and stored at −20°C. The C-genome specific probe (BAC BoB014O06) was used to identify the C-genome chromosomes. The procedures of FISH analyses followed the protocol of Cui et al. (2012) and Zhu et al. (2015).

### RNA extraction, library preparation, and data analysis

Total RNA was isolated with TRIzol reagent (Invitrogen, Life Technologies) following standard protocol from two biological replicates. RNA quality and purity were assessed with the Agilent Technologies 2100 Bioanalyzer (Agilent) according to the RNA Integrity Number (RIN) value. RNA-Seq library construction was processed following TruSeq RNA Sample Prep v2 protocol. Subsequently, the 100 bp paired-end reads were generated via Illumina HiSeq 2000.

We used NGSQCToolkit (v2.3.3) (Patel and Jain, 2012) to check and visualize the quality of the raw data, in order to trim and filter the pair-end reads containing Ns, reads containing adapters, and low quality reads (Reads of low quality base were greater than 20%). Then the clean reads were aligned to the reference genome of *Brassica napus* (Brassica _napus.annotation_v5.gff3.) using HISAT (HISAT version 0.1.6-beta) (Kim et al., 2015) with the default parameters, except for setting the minimum alignment score of L, 0, −0.18. To provide sensitive and accurate results, only unique mapped reads were used in further study. FPKM (Fragments per Kilobase of transcript per Million mapped reads) method was used to predict the gene expression levels (Trapnell et al., 2010).

To study the effects of the genome-dosage regulatory between A- and C-subgenome genes, we used the 31,526 homoeologous gene pairs between A and C subgenomes according to the reference genome sequence data of *B. napus* (Chalhoub et al., 2014). About 54% of these homoeologous gene pairs were removed due to no expression or low expression (FPKM < 1) in diploid parents, finally we selected 14380 homoeologous gene pairs with FPKM values greater than 1.0 in both diploid progenitors. All further analyses were performed on these 14,380 homoeologous gene pairs.

Pearson correlation tests between homoeologous gene expression and genotype dosage (1: 2/3: 1/2:1/2: 1/3) for each A- and C-subgenome gene were performed, respectively. Pearson test and multiple test correction were calculated using adjustment method in R. The P values in the analysis were adjusted for the multiple test correction by the Benjamini–Hochberg method (Benjamini and Hochberg, 1995). Genes with significant expression and dosage correlation (*p* < 0.05) were defined as dosage dependent, whereas genes with no significant expression and dosage correlation were defined as dosage independent.

GO enrichment analysis was performed using Cytoscape plug-in BiNGO (Maere et al., 2005). GO terms with corrected *p* < 0.05 (Benjamini and Hochberg FDR-adjusted P value) were considered to be significantly enriched. The orthologous genes in *A. thaliana* were used to predict the most probable function of the gene pairs as the high homology between *B. napus* and *A. thaliana*. We used the whole *Arabidopsis* genome gene list as background.

### Genes of *cis* and *trans* effects

We measured the *cis*/*trans* effects on gene transcription by comparing the gene transcription difference between A- and C-subgenome genes as defined in previous studies (Tirosh et al., 2009; Shi et al., 2012), briefly as A = log_2_(PA/PC) (both *cis* and *trans* effects), B = log_2_(F_1_A/F_1_C) (*cis* effects) and A − B (*trans* effects) (P = parents, F_1_ = hybrid / polypoid). If A = B and B ≠ 0, genes were classified as “only *cis* effects,” whereas genes were classified as “only *trans* effects” if A ≠ B and B = 0. And if A = B and B = 0, genes were “no *cis*-*trans* effects,” while genes were “*cis*-*trans* effects” if A ≠ B and B ≠ 0. Statistically significant differences were identified using a Fisher's exact test and multiple testing correction (*p* < 0.05).

### Protein interactions for dosage-dependent/-independent genes

The interaction data (Release 3.4.134 compiled on February 25th, 2016) set from BIOGRID (Stark et al., 2006; http://thebiogrid.org/) was used to detect protein–protein interactors for dosage-dependent/-independent genes. The orthologous genes in *A. thaliana* were used to predict the corresponding genes of *Brassica*. Only genes with ≥ 1 interactors were analyzed. The Wilcoxon's rank-sum test was used to test whether the numbers of dosage-dependent/-independent genes were significantly different.

## Results

### Phenotype and cytology of synthesized *B. napus* and derivatives with different genome dosages

The chromosome complements of synthesized *B. napus* hybrid (AC, 2n = 19, A: C = 1: 1) and allotetraploid (CCAA, 2n = 38, A: C = 1: 1), and two derived allotriploids (AAC, 2n = 29, A: C = 2: 1; CCA, 2n = 28, A: C = 1: 2) were confirmed by fluorescence in *situ* hybridization (FISH) analyses with C-genome specific probe (Figure 1C), before they were used to study the gene expressions. While the hybrid and allotetraploid had an intermediate phenotype between two parental diploids, the allotriploid (AAC) was more biased to *B. rapa* and another one (CCA) to *B. oleracea* (Figure 1B), which suggested the dosage effects of the component genomes on the morphological expression.

### Global gene expression levels in synthetic *B. napus* and derivatives

**Figure 2:**
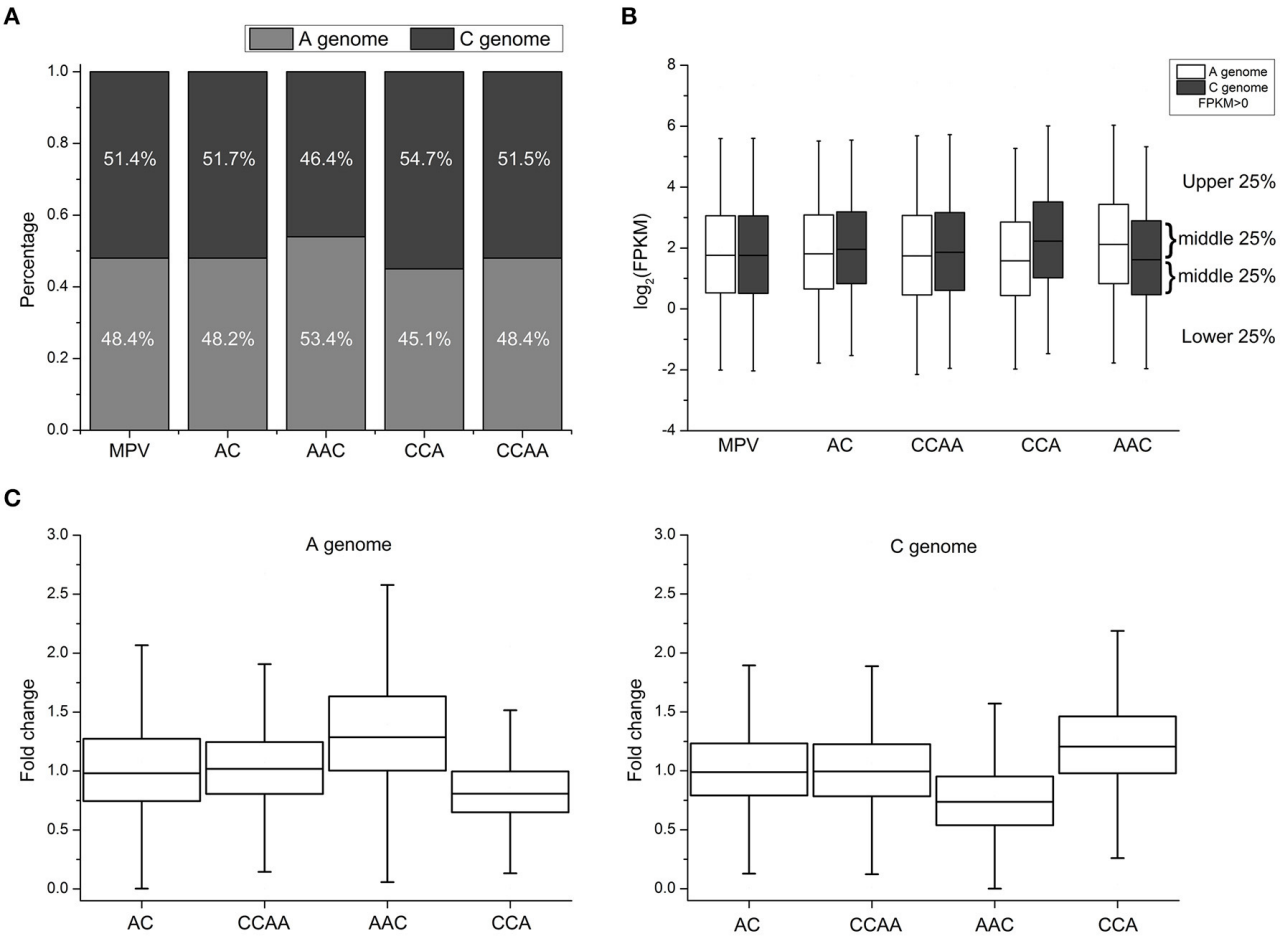
**Gene expression changes in synthetic *B. napus* and derivatives. (A)** The number of expressed genes (FPKM > 0) diversity between A- and C-subgenome genes in MPV, AC, CCAA, AAC, and CCA. **(B)** Global gene expression levels diversity between A- and C-subgenome genes in MPV, AC, CCAA, AAC, and CCA. **(C)** The distribution of fold change (FC) of AC, CCAA, AAC, and CCA, compared with progenitors.

To analyze genome-wide gene expression levels of leaves from these synthetics using RNA-seq, 19–45 million sequencing clean reads were obtained from each of two biological replicates (Table S1). The gene expression levels between two replicates were correlated very well (Average *R* = 0.93, Figure S1). FPKM (Fragments per Kilobase of transcript per Million mapped reads) values were used to represent the gene expression levels (Trapnell et al., 2010). The proportions of the number of expressed genes (FPKM > 0) for A- and C-subgenome were about 48.3 and 51.5% in AC, CCAA, and MPV (mid-parent expression values, representing the progenitors), respectively (Figure 2A, Table S2). The proportions were consistent with those of the reference genome sequence data of *B. napus* (Chalhoub et al., 2014). However, the proportions of A- and C-subgenome genes were significantly different in AAC (Chi-square test, *p* < 2.2e-16; 53.4 and 46.4%, respectively) and CCA (Chi-square test, *p* < 2.2e-16; 45.1 and 54.7%, respectively), in comparison with MPV (Figure 2A, Table S2). In addition, the changes of the proportion between A- and C-subgenome genes were in accordance with dosage alterations, suggesting that dosage variables exert an influence on gene expression.

Then the genome-wide gene expression levels (FPKM > 0) of different materials were investigated, and the results indicated that gene/genome dosage imbalance influenced gene expression levels and global gene expression levels were positively related with genome dosages. As shown in Figure 2B, the global expression level of A-subgenome genes was significantly higher than that of C-subgenome genes in AAC (Wilcoxon signed-rank test, *p* < 2.2e-16), whereas the expression level of A-subgenome genes was significantly lower than that of C-subgenome genes in CCA (Wilcoxon signed-rank test, *p* < 2.2e-16). But there were no significant expression differences between A- and C-subgenome genes in dosage-balance materials (AC and CCAA, A: C = 1: 1) and the parents (MPV, A: C = 1: 1) (Wilcoxon signed-rank test, *p* > 0.05). The results confirmed that gene expression levels could be impacted by genome-dosage balance.

### Expression divergence between *B. napus*/derivatives and parents

To assess what extents of expression levels have changed among materials with different genome dosages, 14,380 homoeologous gene pairs (see Section Materials and Methods) were used to compare expression levels with MPV (mid-parent expression values) calculated by averaging expression values observed in the diploid progenitors. Fold change (FC) was used to quantify the change of expression levels, compared with progenitors. We found that significant differential expressions occurred between A- and C-subgenome genes when the genome dosages varied. The distributions of FC values were almost the same in AC and CCAA with the balanced genome-dosage, and ranged 0.75–1.25 (interquartile range, 0.75–1.25) and the median was about 1.0 (Figure 2C), indicating the genes of both subgenomes expressed highly consistently with those in two parents. Differently in AAC and CCA with the imbalanced genome dosages, the magnitude of the FC values was significantly greater for A-subgenome genes (median = 1.26-fold) than that of C-subgenome genes (median = 0.75-fold) in AAC, while the opposite situation happened in CCA (Wilcoxon's rank-sum test, *p* < 2.2e-16; Figure 2C). Furthermore, the magnitude of FC increased with the proportion of subgenome dosage. In other words, the position of the interquartile range and the median of A-subgenome genes in AAC (approximately 1.0–1.6 and 1.25, respectively) and C-subgenome genes in CCA (approximately 1.0–1.5 and 1.25, respectively) were obviously higher than 1.0 for the median of AC and CCAA. Whereas the interquartile range and the median of C-subgenome genes in AAC (approximately 0.5–1.0 and 0.75, respectively) and A-subgenome genes in CCA (approximately 0.6–1.0 and 0.75, respectively) were definitely less than 1.0 (Figure 2C), suggesting single dosage effects responding to genome-dosage changes. Besides, the genome-wide gene expression levels of AC and CCAA were almost the same, for no significant difference was detected (Wilcoxon's rank-sum test, *p* > 0.05; Figure 2B). The observation supported the view that the effect of dosage was not simply due to the absolute dosage of the copy number, but rather resulted from a change in the relative dosage balance between A and C subgenomes (Birchler et al., 2005; Bekaert et al., 2011).

There was also dosage compensation at the mRNA level. We found that it was not the expected 2-fold change in gene expression levels along with the change of subgenome ratio (A/C = 2) in AAC. Data showed that the positions of the interquartile range and the median of A-subgenome genes were about 1.0–1.6 and 1.25, respectively, but were about 0.5–1.0 and 0.75 for C-subgenome genes (Figure 2C). It was similar in CCA. In other words, genes expressed did not show the expected 2-fold change in gene expression levels if mRNA levels correlated perfectly to gene/genome dosage, which suggested that there were buffering effects or dosage compensation at the mRNA level. Some clues could also be revealed from the global gene expression levels (Figure 2B). The results collectively indicated a complex relationship between gene dosage and expression.

### Correlations between gene expression and dosage

**Figure 3:**
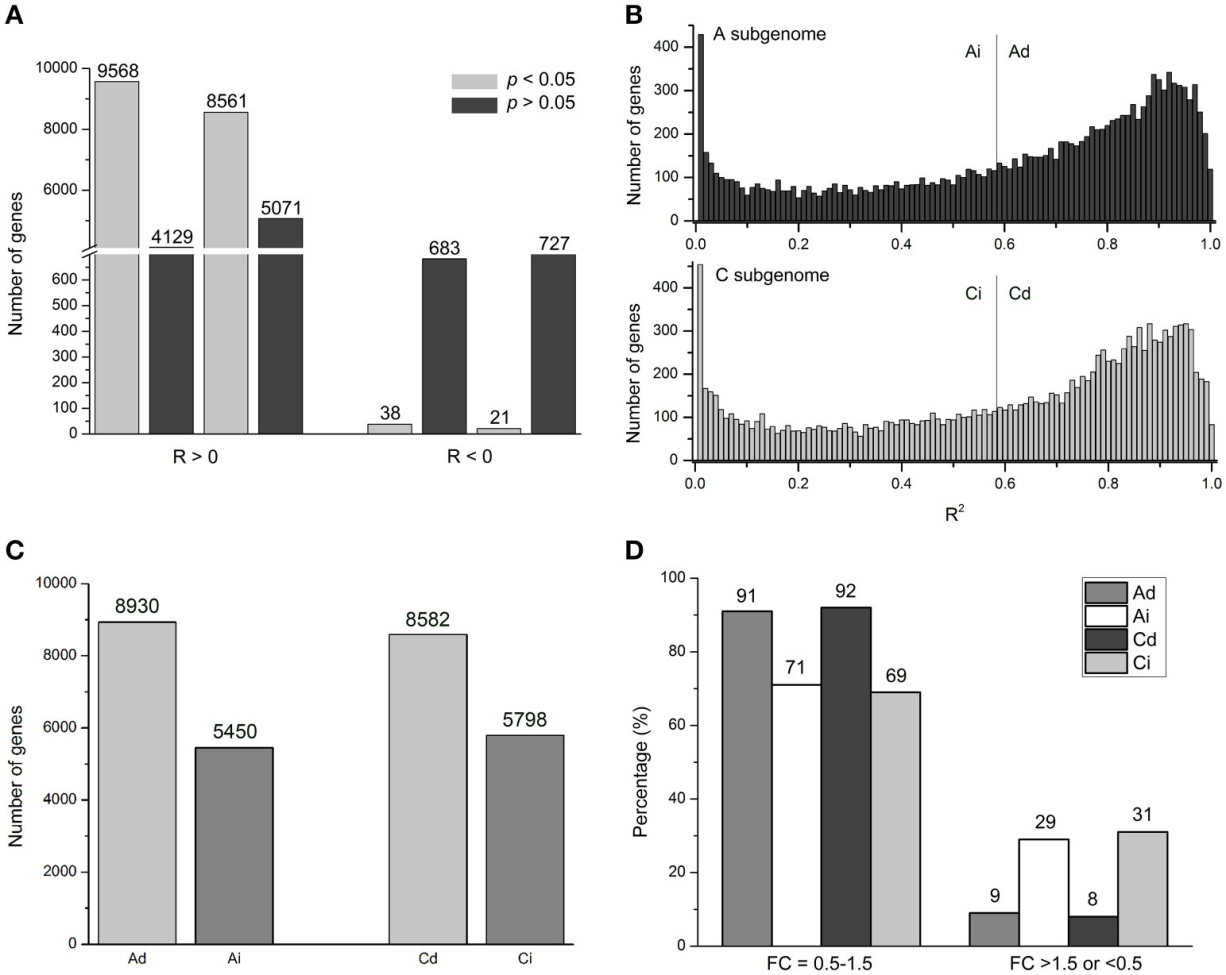
**Genome-wide dosage regulation of homeologous expression in synthetic *B. napus*. (A)** Number of genes with positive (*R* > 0) and negative (*R* < 0) correlation between dosage and expression of A (left) and C (right) homeologous genes at significant (FDR < 0.05) and insignificant (FDR > 0.05) levels. **(B)** The distribution of coefficient of determination (*R*^2^) in A and C homeologous. X axis: *R*^2^ value bins divided; Y axis: number of genes in each bin. Gray lines separate genes with dosage-dependent and dosage-independent expression. **(C)** Number of Ad, Ai, Cd, and Ci genes. **(D)** Numbers of genes with fold-change (FC) distribution of in A and C homeologous genes at “0.5–1.5” and “>1.5 or <0.5” levels in CCAA. Ad, Ai, Cd, and Ci means dosage dependent A, dosage independent A, dosage dependent C, and dosage independent C, respectively.

To study correlations between gene expression and dosage, we calculated the correlation coefficient (*R*-values) between homoeologous gene expression levels (FPKM) and relative dosages of the 14,380 homoeologous gene pairs between A and C subgenomes, respectively. For example, for gene *BnaA01g05230D* with *R* = 0.97, an A-subgenome gene, expression levels of AA (all A genome), AAC (2/3 A genome), AC (1/2 A genome), CCAA (1/2 A genome), and CCA (1/3 A genome) were 22.22, 11.37, 7.41, 8.98, and 6.34, respectively. Pearson correlation between expression levels (22.22, 11.37, 7.41, 8.98, and 6.34) and the relative dosage (1, 2/3, 1/2, 1/2, and 1/3) was 0.97 (Table S3). Results showed that about 95% *R*-values were > 0, meaning positive correlations, and the remaining 5% *R*-values were < 0 for negative correlation (Figure 3A, Table S3). Moreover, among those 5% genes with *R* < 0, only 38 A-subgenome genes and 21 C-subgenome genes (0.26 and 0.15%, respectively) had statistical significance. While most genes (average 63%) were statistically significant among those genes with *R* > 0 (Figure 3A, Table S3). We concluded that the majority of genes showed positive correlations between gene expression and genome dosage in *Brassica* polyploids and hybrids.

The small group of negatively correlated genes which were called “inverse dosage effect” genes (Birchler and Veitia, 2012; Veitia et al., 2013) could be potentially interesting, due to their negative effect of the regulator to cancel the positive effect of the change in gene dosage on the total expression levels. In the following, we investigated the molecular function and biological pathway of the potentially small group of significantly inverse dosage effect genes with the DAVID Functional Annotation Tool (https://david.ncifcrf.gov/tools.jsp). After the orthologous genes in *A. thaliana* of those negatively correlated genes were submitted to the DAVID, by using the *Arabidopsis* genome gene list as background, the genes involved in proteolysis and/or macromolecule catabolic process (GO: 0006508, GO: 0009057) were highestly over-represented, followed by those for translation and/or ribosome (GO: 0006412, GO: 0005840; Table S4).

### Dosage-dependent and -independent genes expressions

In order to study the effects of the relative dosage balance, coefficients of determination (*R*^2^) between dosage and expression level were used to quantify the strength of dosage effects on expression. High *R*^2^-values indicated that the gene expression levels and dosage changes were strongly correlated, and the corresponding genes were defined as dosage dependent. On the contrary, genes with low *R*^2^-values were called dosage-independent, suggesting that the expression level was weakly correlated with the dosage changes. For example, for dosage dependent *BnaA10g21130D*, an A-subgenome gene with *R*^2^ = 0.9996, expression levels were 6.32: 3.96: 2.87: 2.85: 1.69, close to 1: 2/3: 1/2: 1/2: 1/3. In contrast, for dosage independent gene *BnaC05g12440D* (*R*^2^ = 2.39E-9), the gene expression levels were 96.35: 71.47: 94.49: 71.31: 99.86, far from 1: 2/3: 1/2: 1/2: 1/3 (Table S3).

All genes were clustered into two groups based on *R*^2^-values using the Pearson correlation test with multiple testing correlation (Benjamini and Hochberg, 1995), as defined in *Arabidopsis* (Shi et al., 2015). Empirically *p* < 0.05 was used as the cutoff value for the statistically significant level. Maintaining the significant level at 0.05 in both A- and C- subgenome genes, we got *R*^2^ > 0.59. So those genes with *R*^2^ > 0.59 were called dosage dependent A (Ad) and C (Cd) genes, while others with *R*^2^ < 0.59 were called dosage independent A (Ai) and C (Ci) genes (Figure 3B, Table S3). However, the distribution was continuous, there was no obvious boundaries between dosage dependent and independent genes. Under this cutoff value, about 60% genes (8930 and 8582 genes in A and C subgenomes, respectively) were dosage dependent, and the rest of 40% genes (5450 and 5798, respectively) were dosage independent (Figure 3C, Table S3).

Dosage dependent and independent genes definitely influenced the strength of dosage effects on gene expression. In CCAA, the FC values of most Ad and Cd genes (91 and 92%, respectively) distributed in “0.5–1.5” compared with MPV, significantly greater than those of Ai and Ci genes (71 and 69%, respectively; Chi-square test, *p* < 2.2e-16). On the contrary, less than 10% of genes appeared in the regions with FC values “> 1.5 or < 0.5” of Ad and Cd, whereas 30% of Ai and Ci genes were significantly higher (Chi-square test, *p* < 2.2e-16; Figure 3D). Dosage dependent and independent genes contributed differentially to variable gene expressions, likely the former to additive expression and the latter to non-additive expression.

### Biological characteristics of dosage-dependent and -independent genes

**Figure 4:**
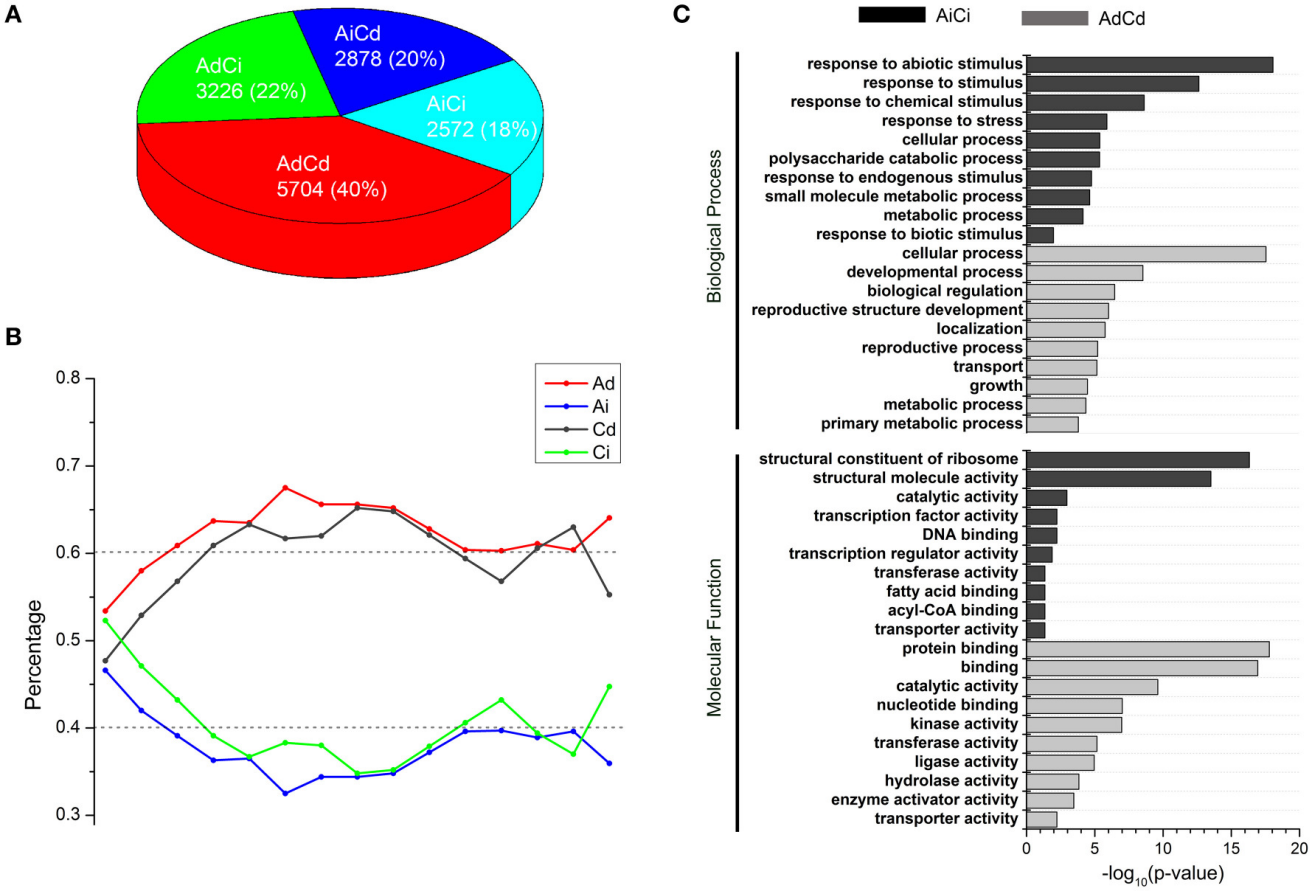
**Dosage-dependent and dosage-independent expressions of homeologous in *B. napus*. (A)** Number and percentage of AdCd, AdCi, AiCd, and AiCi genes. **(B)** Percentages of Ad (red), Ai (blue), Cd (black), Ci (green) genes that contain absolute expression levels (FPKM) within a range of distances. X axis: a range of bins contains FPKM values ranking from low to high; Y axis: percentage of genes in each bin. Dash lines represent the expected levels. **(C)** Enrichment of AdCd and AiCi genes in GO groups. Ad, Ai, Cd, and Ci represent dosage dependent A, dosage independent A, dosage dependent C, and dosage independent C, respectively.

A total of 14,380 homeologous gene pairs were classified into four groups: AdCd (40%), AdCi (22%), AiCd (20%), and AiCi (18%) (Figure 4A, Table S3), which was not significantly different from the expected percentages of AdCd (37%), AdCi (25%), AiCd (23%), and AiCi (15%) (Chi-square test, *p* > 0.05). The similar dosage dependency of both A- and C-subgenome genes suggested possible generalities for factors controlling which genes were dosage dependent or independent. So genes in groups of AdCd and AiCi definitely represented two opposite types divided by whether the gene expression was dependent or independent of the dosage changes.

As the gene expression was either dosage dependent or independent, we wanted to know if the absolute expression levels of genes worked. We divided the absolute expression levels ranking from low to high into “bins,” each containing 1000 genes and counted the number of dosage dependent (Ad, Cd) and dosage independent (Ai, Ci) genes in each bin. The distribution of dosage-dependent and -independent genes in bins with different absolute expression levels had no significant differences than the expected (about 60 vs. 40%) in CCAA (Chi-square test, *p* > 0.05; Figure 4B), indicating that the absolute expression levels of dosage-dependent genes did not differ from those of dosage-independent genes. Therefore, the absolute expression levels were not the determining factor of dosage dependence and/or independence.

Gene ontology (GO) enrichment analysis was used to classify genes according to their molecular function and the pathway in which they were involved. In this study, the orthologous *A. thaliana* Gene ID was used to predict the corresponding genes in *B. napus*. GO analysis revealed different functional enrichment between genes with dosage dependent and independent. First, the top 3000 of AdCd genes were submitted to BiNGO (Maere et al., 2005), and they were significantly enriched (*p* < 0.05) in lots of categories, including the most basically process, like “cellular process,” “developmental process,” “biological regulation,” “localization,” “transport,” etc. (Figure 4C, Table S5). However, AiCi genes were mainly clustered into the GO terms of “response to stimulus” in biological process, which consisted of a mass of daughter categories, such as “response to stress,” “response to abiotic stimulus,” “response to chemical stimulus” and so on (Figure 4C, Figure S2, and Table S5). So the dosage independent genes probably played a key role in stress responses. The other main modules contained the GO terms including “structural constituent of ribosome,” “DNA binding” and “transcription factor activity” in molecular function (Figure 4C, Table S5).

### *cis* and *trans* effects on gene expression of dosage-balance regulatory

**Figure 5:**
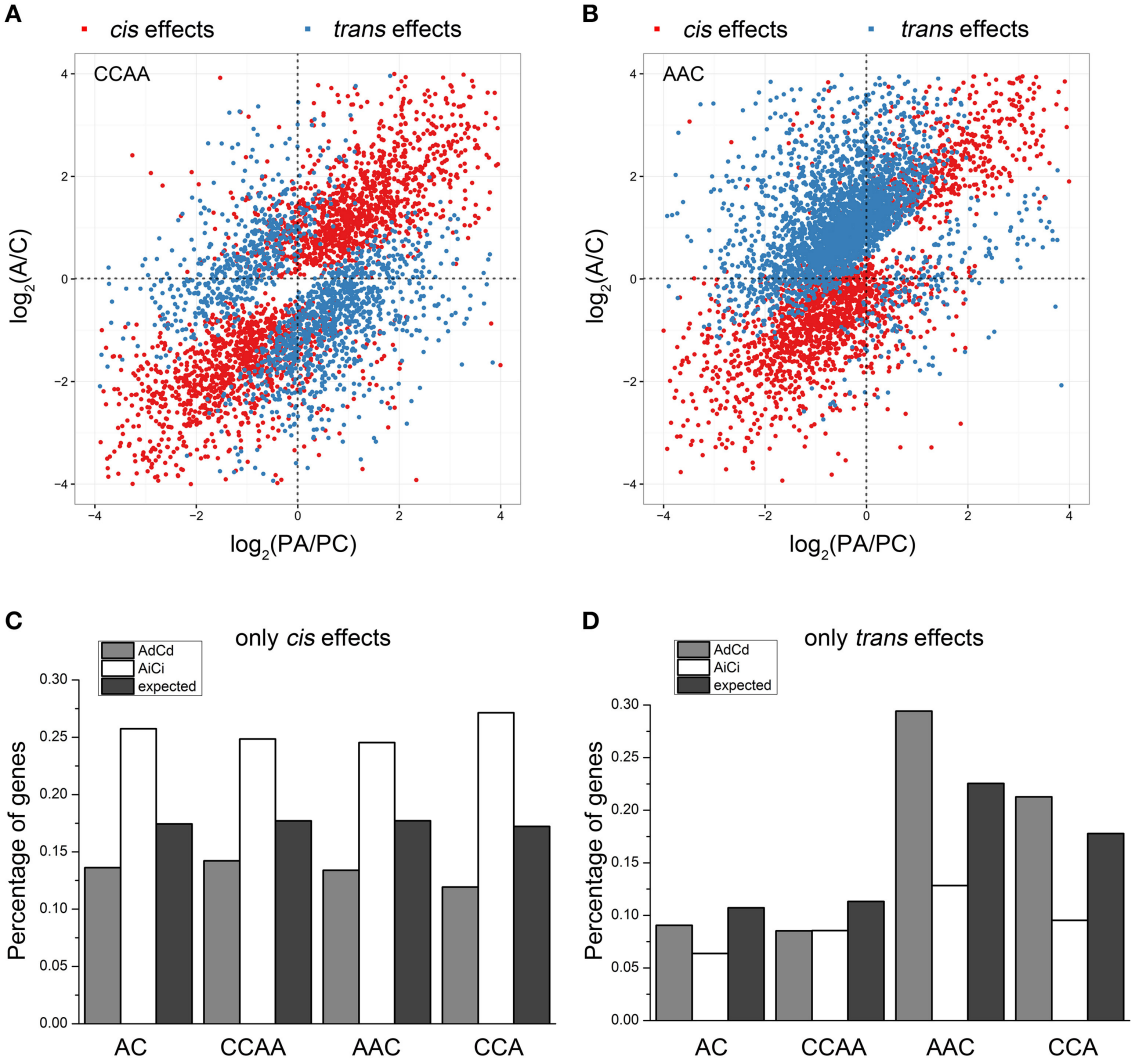
***Cis* and *trans* effects on gene expressions of dosage-balance regulatory. (A,B)** The distributions of “*cis* effects” and “*trans* effects” in CCAA and AAC, respectively. **(C,D)** The percentages of “only *cis* effects” and “only *trans* effects” diversities between A and C homeologous genes in AC, CCAA, AAC, and CCA. Ad, Ai, Cd, and Ci means dosage dependent A, dosage independent A, dosage dependent C and dosage independent C, respectively.

Gene expression changes in gene transcription could result from *cis* and/or *trans* effects (Rockman and Kruglyak, 2006; Williams et al., 2007; Yoo et al., 2014). In this study, we measured the *cis*/*trans* effects on gene transcription by comparing the transcription difference between A- and C-subgenome genes (see Section Materials and Methods), as defined in other studies (Tirosh et al., 2009; Shi et al., 2012). Results showed almost the same gene distribution of “only *cis* effects,” “only *trans* effects,” “*cis*-*trans* effects,” and “no *cis*-*trans* effects” in dosage-balanced AC and CCAA. However, the gene percentage of *trans* effects differed significantly in AAC (22.5%) and CCA (17.8%), compared with AC (10.7%) or CCAA (11.3%) (Chi-square test, *p* = 4.395e-11), whereas *cis* effects remained the same (Chi-square test, *p* = 0.2727), approximately 17.5% (Figures 5A,B, Figure S3, and Table 1). It was shown that *trans* effects rather than *cis* effects were mainly responsible for gene/genome dosage variations.

**Table 1:** **The distribution of *cis* and *trans* effects in synthetic *B. napus* and derivatives**.

**Materials**	**No effects**	**Only *cis* effects**	**Only *trans* effects**	***Cis*-*trans* effects**
AC	9688 (67.4%)	2507 (17.4%)	1541 (10.7%)	644 (4.5%)
AAC	7485 (52.1%)	2547 (17.7%)	3242 (22.5%)	1106 (7.7%)
CCA	8688 (60.4%)	2476 (17.2%)	2557 (17.8%)	659 (4.6%)
CCAA	9436 (65.6%)	2546 (17.7%)	1627 (11.3%)	771 (5.4%)

Then we wanted to know if dosage-balance regulatory influenced gene expression associated with *cis*/*trans* effects. We explored the distribution of dosage-dependent (AdCd) and -independent (AiCi) genes in groups of “only *cis* effects” and “only *trans* effects.” For *cis* effects, the distribution of AiCi genes was significantly greater than expected (Chi-square test, *p* < 0.01), whereas that of AdCd genes was significantly less than expected (Chi-square test, *p* < 0.01; Figure 5C), indicating that dosage-independent genes more likely influenced expression by *cis* effects. Inversely, dosage-dependent genes were more inclined to impact expression by *trans* effects, for the data showed that the distribution of AdCd genes was significantly greater than expected (Chi-square test, *p* < 0.01), while that of AiCi genes was significantly less than expected (Chi-square test, *p* < 0.01) for *trans* effects in dosage-imbalance group (Figure 5D).

### Protein interactions for dosage-dependent and -independent genes

**Figure 6:**
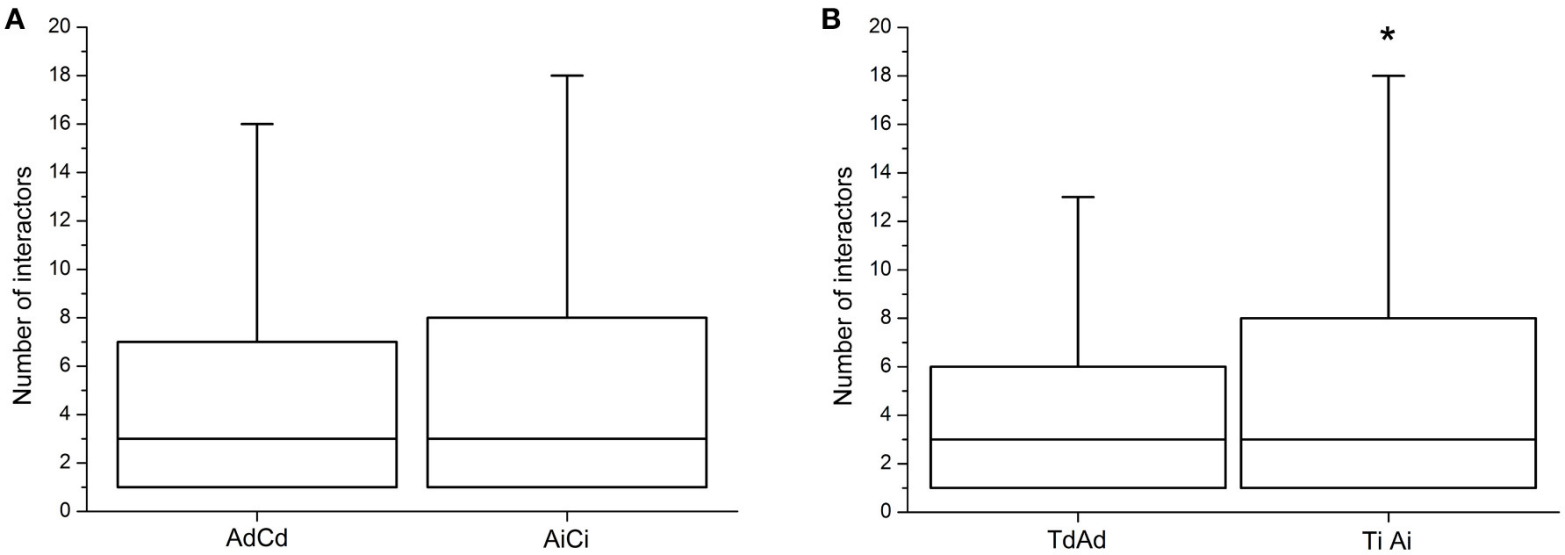
**The degrees of protein interactions for dosage-dependent and dosage-independent genes. (A)** The number of protein interactions for dosage-dependent and dosage-independent in AdCd and AiCi of *B. napus*. **(B)** The number of protein interactions for dosage-dependent and dosage-independent in TdAd and TiAi of *Arabidopsis*. Dosage-independent genes have significantly more protein interactions. Asterisks indicate *p* < 0.05. Ad, Ai, Cd, and Ci means dosage dependent A, dosage independent A, dosage dependent C, and dosage independent C, respectively.

The dosage balance hypothesis stated that dosage imbalance of subunits of macromolecular complexes could affect the eventual amount of complexes formed (Birchler et al., 2005; Veitia et al., 2008). Liang et al. (2008) predicted that the duplicates of a highly under-wrapped protein should be more sensitive to dosage imbalance. This meant that dosage-dependent genes might be expected to have fewer protein–protein interactions than dosage-independent ones. From the investigations of the number of interactors of dosage-dependent/-independent genes, the dosage-dependent genes (AdCd) had lower number of protein–protein interactions than that of dosage-independent (AiCi), but the difference was insignificant (Wilcoxon's rank-sum test, *p* = 0.2302; Figure 6A), possibly due to the lack of reference data and the use of the orthologous genes in *A. thaliana* to predict the corresponding gene in *Brassica*. By using previously published data in *Arabidopsis* (Shi et al., 2015) and reanalysis, it was found that the dosage-independent genes (TiAi) had a significant greater number of protein–protein interactions than that of dosage-dependent (TdAd) (Wilcoxon's rank-sum test, *p* = 0.04692; Figure 6B).

## Discussion

### The impacts of gene/genome dosage changes on gene expression

Gene copy number changes generally translate into changes in gene expression, which was supported by lots of cases in yeast, mouse, human and *Arabidopsis* (Tang and Amon, 2013). We also found gene expression changes caused by gene dosage or copy number alterations (Figure 2), together with phenotypes alteration driven by the cumulative effects of dosage changes of a large number of genes (Figure 1). Recent works have shown that additive expression was the prevailing gene expression pattern when two parental genomes were present in an allopolyploid nucleus, although non-additively expressed genes represented a small portion (Wang et al., 2006; Chagué et al., 2010; Jiang et al., 2013; Yoo et al., 2013; Zhao et al., 2013). In our results, many genes generally increased their expressions along with the increase of gene/genome dosage (Figure 2), and were defined as dosage dependent, for their expressions showed well correlations with dosage changes. However, some genes defined as dosage independent did not exhibit the simple additivity that was predicted to be buffered, because their expressions had poor correlation with dosage changes. Gene dosage balance was one of the important factors for non-additive gene expression in polyploids, although our understanding remained limited (Yoo et al., 2014).

In our study, both A and C subgenomes genes were subjected to dosage dependent and independent expression regulations in synthetic *B. napus*, which represented approximately 60 and 40%, respectively. About 58% of A and C-subgenome genes were in the same direction to either dosage-dependent (40% AdCd) or dosage-independent expression (18% AiCi), while 42% of genes (22% AdCi and 20% AiCd) were in different directions (Figure 4A). In comparison with the results of TdAd (54%), TdAi (15%), TiAd (13%), and TiAi (17%) from *Arabidopsis* (Shi et al., 2015), our data indicated a little different percentage distribution. As to the reasons for the difference, the first one might be the different cutoff values used to distinguish dosage-dependent and -independent genes, as there was no clear distinction between them. The second was perhaps the different genetic backgrounds between *Arabidopsis* and *Brassica* species, as diploid *Brassica* genomes were triplicated compared with *A. thaliana*, as confirmed by recent whole genome sequencing of *B. rapa* (Wang et al., 2011), *B. oleracea* (Liu et al., 2014; Parkin et al., 2014), and *B. napus* (Chalhoub et al., 2014). Thereafter along with some losses of duplicated copies from the genomes during evolution, it was more complex for gene-dosage alterations in *Brassica*.

Gene expression changes in transcription could result from *cis* and/or *trans* effects (Rockman and Kruglyak, 2006; Williams et al., 2007; Yoo et al., 2014). It was previously suggested that *trans*-acting factors played a much larger role than *cis*-factors in causing gene expression variation between different species (Dong et al., 2011; McManus et al., 2014; Wang et al., 2015). Our results also showed that *trans* effects played a key role in response to gene/genome dosage alterations (Table 1). Besides, we observed that dosage-dependent genes more likely impacted expressions by *trans* effects (Figure 5D), whereas dosage-independent genes were more inclined to influence expression by *cis* effects (Figure 5C). The *cis* and *trans* regulatory factors differed in influencing the evolution of gene regulation, *cis*-effect regulatory affected the expression of nearby genes (e.g., changes in promoters and enhancers) on the same chromosome (Wittkopp et al., 2008; Tirosh et al., 2009; Dong et al., 2011; Wittkopp and Kalay, 2012). Thus, the regulation via many complex subunits possibly explained why *cis* effects overrepresented in dosage-independent genes. But *trans*- effect regulatory impacted both alleles of the diploid progenitors, dosage variations of alleles belonging to dosage-dependent genes most likely regulated expression via *trans* effects.

### Dosage compensation for gene/genome dosage changes on gene expression

Dosage compensation was a common biological phenomenon that was clearly supported by many cases in a wide range of organisms, including *Drosophila*, mouse, human, maize, etc. (Veitia and Potier, 2015). The most typical example was X chromosome inactivation in mammals, which was a mechanism that equalized the number of active X chromosomes in eutherian females (XX) and males (XY) (Pessia et al., 2012; Veitia and Potier, 2015). In our study, we also found that there were not only clear dosage effects of gene expression responding to gene/genome dosage changes, but also obviously dosage compensation effects. As showed in Figure 2, the genes expressed did not show the expected 2-fold change in their expression levels if mRNA levels correlated perfectly to gene/genome dosage in AAC and CCA, suggesting dosage compensation at the mRNA level.

Veitia et al. (2013) suggested that the inverse dosage effect genes or negatively operated dosage effectors could lead to dosage compensation, as the positive effect of the dosage changes on expression was canceled by the negative effect of the regulator. In our results, some inverse dosage effect genes were detected (Figure 3A). Such genes with statistical significance involved in proteolysis (GO: 0006508) and ribosome (GO: 0005840) were highly over-represented (Table S4), and might contribute to dosage compensation. The result was consistent with previous study that proteolysis was predicted to play a crucial role in buffering the effects of gene dosage alterations (Veitia et al., 2008; Lundberg et al., 2012; Veitia and Potier, 2015), as induction proteolysis appeared to be a general response to the genomic imbalance due to aneuploidy (Lundberg et al., 2012). The cell might increase the level or activity of proteolysis and/or chaperones to cope with the overexpression of hundreds of proteins, many of which belonged to complexes, caused by the presence of supernumerary chromosomes (Veitia and Potier, 2015). In addition, the result that ribosome (GO: 0005840) was highly represented in the inverse dosage effect genes was consistent with a frequent biological phenomenon of nucleolar dominance, a non-additive or uniparental expression of rRNA genes, observed in many allopolyploids including *B. napus* (Chen and Pikaard, 1997).

Furthermore, we found that dosage-independent genes were more likely buffered for response to dosage changes than dosage-dependent genes (Figure 2C), which coincided with the result that the greater number of protein–protein interactions was investigated in dosage-independent genes (Figure 6B), suggesting that the existence of dosage-balanced multi-subunit complexes or networks contributed to dosage compensation. Indeed, such findings were reported in *Drosophila* (Zhou, J. et al., 2011; Malone et al., 2012). Changes in gene copy number resulted in changes of protein levels in the majority of cases in aneuploid budding yeast and human cells by quantitative proteomic analyses (Pavelka et al., 2010; Torres et al., 2010; Stingele et al., 2012), while the proteins that did not show the coordinated increase with gene copy number were found to be predominantly components of large protein complexes (Torres et al., 2010; Stingele et al., 2012). This was possibly explained by the dosage balance hypothesis (Veitia et al., 2008, 2013; Birchler and Veitia, 2010) that the co-variation of a target gene along with a linked controlling gene could lead to dosage compensation at the transcriptional level.

### Dosage-balance influence in regulatory evolution

Dosage imbalance could influence protein complexes that resulted in novel phenotypes, leading to great variability for adaptations in evolution (Birchler et al., 2005; Veitia et al., 2008; Veitia and Potier, 2015). In our GO enrichment analysis (Figure 4C, Table S5), dosage-dependent genes were mainly associated with the basic biological processes, although Lloyd et al. (2014) suggested an exceptive example that meiosis genes were often maintained in single copy per genome, revealing that these genes were important to maintain growth and developmental stability, and might provide genetic stability against null mutations and selective advantage by dosage-dependent gene regulation (Ha et al., 2007). In other side, dosage-independent genes were more likely enriched in stress response related processes, suggesting that these genes might accelerate fitness and adaptation. Furthermore, the fact that dosage independent genes with Gene Ontology terms associated with response to important environmental factors, transcription factors and ribosomal protein genes was in accordance well with the fact that genes were most likely retained in WGD duplicates in *B. rapa* (Wang et al., 2011). This result was often explained by the gene dosage hypothesis in which genes encoding products that interacted with one another should be over retained and genes with products that did not interact with other gene products should be lost (Birchler et al., 2005; Freeling and Thomas, 2006; Birchler and Veitia, 2010; Wang et al., 2011; McGrath et al., 2014). It was similar to previous report of Bekaert et al. (2011) that constraints on the relative dosages of central network genes represented an important force for maintaining duplicates. Constraints on dosage-balance over time could result in preferential rewiring of certain biological pathways to execute novel functionality (De Smet and Van de Peer, 2012), and likely helped them to cope with new ecological opportunities and/or challenges (Schranz et al., 2012; Fawcett et al., 2013).

## Conclusion

The gene expression divergence and various phenotypes in the serial *Brassica* polyploids were attributable to gene/genome dosage effects and/or dosage compensations which were correlated with the expressions of dosage-dependent/-independent genes. Those dosage-dependent genes affected expressions more by *trans* effects, and dosage-independent genes acted more by *cis* effects. While the *trans* effects were more responsible for total gene expression changes responding to dosage alterations. Furthermore, dosage-dependent genes were mainly associated with the basic biological processes, whereas dosage-independent genes were more involved in the stress response processes. Future studies should focus on the genetic and epigenetic mechanisms behind the gene expression changes and the related phenotypic changes associated with dosage imbalance.

## Author contributions

ZL conceived the experiment. CT, QP performed the research. CT, CC, and YX produced the materials used. CT, QP, and XG contributed to data analysis, bioinformatics analysis. CT, ZL wrote the manuscript. All authors reviewed and approved this submission.

## Availability of supporting data

All the sequencing data used in this research have been submitted to public database GEO under GSE81845. Other supporting data are included within the article and its additional files.

### Conflict of interest statement

The authors declare that the research was conducted in the absence of any commercial or financial relationships that could be construed as a potential conflict of interest.

## Supplementary material

Figure S1 **Scatter plots of correlations between two independent biological replicates**.

Figure S2 **The majority of AiCi genes enriched in stress related pathway**.

Figure S3 **The distribution of “*cis* effects” and “*trans* effects” in AC and CCA, respectively**.

Table S1 **Summary of reads mapped to *B. napus* reference genome**.

Table S2 **Number and percentage of expressed genes in synthetic *B. napus* and derivatives**.

Table S3 **Correlations between genes dosage and expression (FPKM)**.

Table S4 **Function annotations of inverse dosage effect genes**.

Table S5 **Function annotations of AdCd and AiCi genes**.
