# Supplementary figures and images for: Genome-Wide Gene/Genome Dosage Imbalance Regulates Gene Expressions in Synthetic Brassica napus and Derivatives (AC, AAC, CCA, CCAA)
Source: Front Plant Sci. 2016 Sep 23;7:1432. doi: 10.3389/fpls.2016.01432 (PMC5033974; doi:10.3389/fpls.2016.01432)

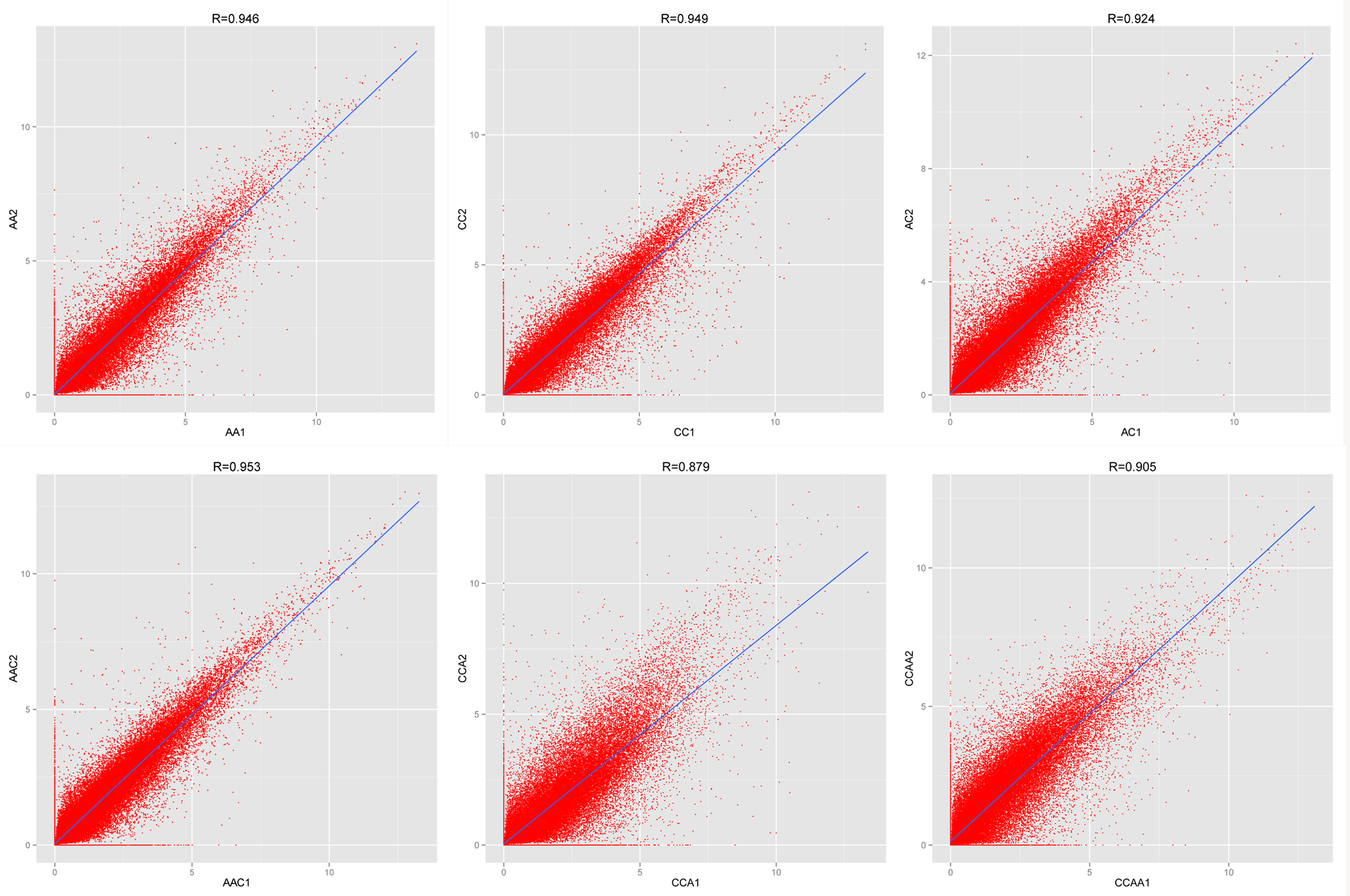

Supplement: Figure S1 — Scatter plots of correlations between two independent biological replicates. [file Image1.TIF]

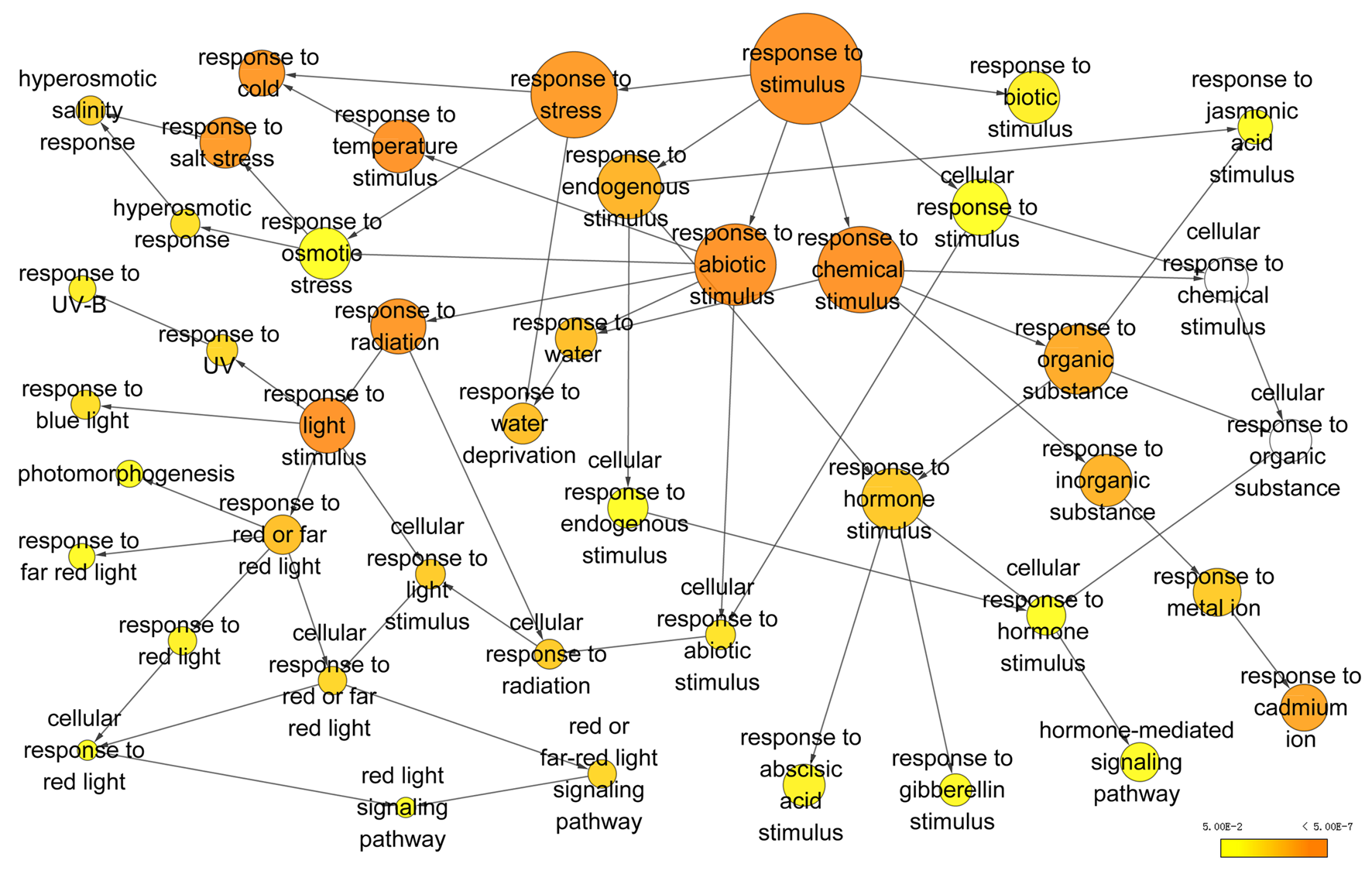

Supplement: Figure S2 — The majority of AiCi genes enriched in stress related pathway. [file Image2.TIF]

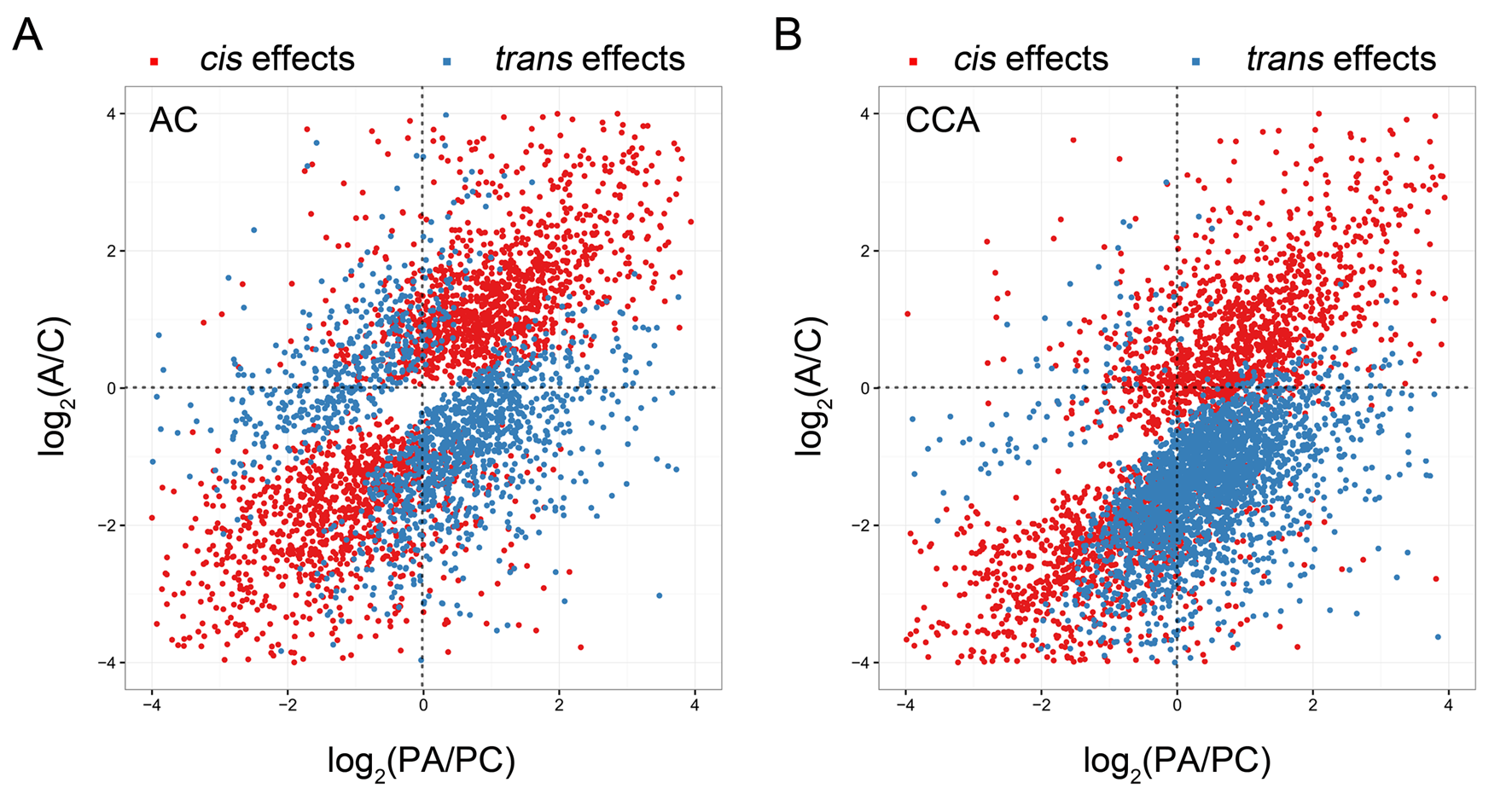

Supplement: Figure S3 — The distribution of “cis effects” and “trans effects” in AC and CCA, respectively. [file Image3.TIF]
