# Supplementary material for: Genome-Wide Gene/Genome Dosage Imbalance Regulates Gene Expressions in Synthetic Brassica napus and Derivatives (AC, AAC, CCA, CCAA)
Source: Front Plant Sci. 2016 Sep 23;7:1432. doi: 10.3389/fpls.2016.01432 (PMC5033974; doi:10.3389/fpls.2016.01432)
Supplement: Table S1 — Summary of reads mapped to B. napus reference genome. [file Table1.DOCX]

**Table S1. Summary of reads mapping to *B. napus* reference genome.**

| **Samples** | **Clean reads** | **Total mapped** | **Uniquely mapped** |
| --- | --- | --- | --- |
| **AA1** | 24693186 | 18931095 (76.67%) | 17689794 (71.64%) |
| **AA2** | 19181688 | 14538126 (75.79%) | 13534102 (70.56%) |
| **CC1** | 22279284 | 16590026 (74.46%) | 15508672 (69.61%) |
| **CC2** | 26422716 | 20028341 (75.80%) | 18779814 (71.07%) |
| **AC1** | 29255432 | 21932118 (74.97%) | 20365770 (69.61%) |
| **AC2** | 25426918 | 19402349 (76.31%) | 18100084 (71.18%) |
| **AAC1** | 30013276 | 22889006 (76.26%) | 21288742 (70.93%) |
| **AAC2** | 32433276 | 24920047 (76.83%) | 23229770 (71.62%) |
| **CCA1** | 30301126 | 23080985 (76.17%) | 21615314 (71.34%) |
| **CCA2** | 26951680 | 20711632 (76.85%) | 19311788 (71.65%) |
| **CCAA1** | 42703426 | 32655699 (76.47%) | 30569124 (71.58%) |
| **CCAA2** | 45697134 | 34881027 (76.33%) | 32631846 (71.41%) |

**Table S2. Number and percentage of expressed genes in synthetic *B. napus* and derivatives.**

| **Materials** | **A-subgenome genes** | **C-subgenome genes** | **Total** |
| --- | --- | --- | --- |
| **MPV** | 19843 (48.4%) | 21080 (51.4%) | 40990 |
| **AC** | 17241 (48.2%) | 18499 (51.7%) | 35793 |
| **AAC** | 19292 (53.4%) | 16764 (46.4%) | 36108 |
| **CCA** | 16454 (45.1%) | 19942 (54.7%) | 36455 |
| **CCAA** | 19523 (48.4%) | 20790 (51.5%) | 40378 |
